# A New Proposal for Soybean Plant Stand: Variation Based on the Law of the Minimum

**DOI:** 10.3390/plants13223193

**Published:** 2024-11-14

**Authors:** Fábio Henrique Rojo Baio, Ricardo Gava, Larissa Pereira Ribeiro Teodoro, Rita de Cássia Félix Alvarez, Marcos Eduardo Miranda Alves, Dthenifer Cordeiro Santana, Cid Naudi Silva Campos, Ana Carina da Silva Cândido, Paulo Eduardo Teodoro

**Affiliations:** Agronomy Department, Universidade Federal de Mato Grosso do Sul, Rodovia MS 306, km. 305, Caixa Postal 112, Chapadao do Sul 79560-000, MS, Brazil; fabio.baio@ufms.br (F.H.R.B.); ricardo.gava@ufms.br (R.G.); larissa_ribeiro@ufms.br (L.P.R.T.); rita.alvarez@ufms.br (R.d.C.F.A.); marcos.eduardo@ufms.br (M.E.M.A.); dthenifer.santana@ufms.br (D.C.S.); cid.campos@ufms.br (C.N.S.C.); ana.candido@ufms.br (A.C.d.S.C.)

**Keywords:** precision agriculture, glycine max, spatial variability, variable rate seeding, Liebig’s law

## Abstract

The hypothesis of this study is that it is possible to determine the plant stand in the soybean (*Glycine max* L. Merril) crop based on the spatial variability of management units, which are limiting factors in maximizing crop yield. Our objectives were as follows: (I) to evaluate the relationship between soil physical and chemical attributes to establish potential management units for variable-rate seeding; (II) to propose a method for varying plant stands based on the law of minimum soil nutrients; an (III) to relate the interaction between different plant stands on soybean grain yield, taking into account the interaction between the spatial variability of the mapped attributes. Field experiments were carried out on two plots over two agricultural years. The areas were seeded by randomly varying the soybean stand across strips in the first year. The most limiting soil nutrient was established and used, together with the soil CEC, to determine management units (MUs), which were also used to seed soybeans in VRT (Variable Rate Technology) in the same plots in the second year. MUs with the lowest restriction for maximizing yield were sown in the second year with the lowest plant stand. Data were processed using multivariate statistics. Our findings reveal that it is possible to establish MUs for seeding soybeans with different stands following the spatial variability of limiting soil nutrients according to the law of the minimum and thus increase the crop grain yield. Spatial variability of potassium (K) in the plot, identified as limiting, affected the spatial variability of grain yield. Decreasing plant stands in MUs with the lowest limitation level increases yield. However, increasing the stand in MUs with a higher limitation level can lead to increased intraspecific competition, affecting yield as well as increasing input costs.

## 1. Introduction

Soybean [*Glycine max* (L.) Merril] variable rate seeding technology (VRT) using principles of spatial variability in precision agriculture can be carried out by several automated seeders already available on the market, incorporated with electronic controllers to vary the rotation of the seed dispensing disks, varying the plant stand based on a prescription map [1]. However, recommendations for setting different soybean populations on the same plot still need further field trials [2].

Seeding rates with changes in seed population influence the growth and development of soybean plants, as well as affecting production costs [3]. Soybean sowing rate can also influence the severity of a fungal disease attack or weed infestation [4].

Plant architecture is determined by genetics but can be influenced by environmental conditions. This architecture is important for capturing sunlight, which can positively or negatively affect the photosynthetic capacity of the plant canopy [5]. Increased soybean stand leads to higher intraspecific competition for the water available in the soil [6], leading to an allocation of dry matter to the shoot and root, which in turn promotes an improved competition capacity for resources such as water and nutrients [7,8]. Thus, varying the soybean stand has an effect on its physiology, influencing the formation of branches and directly affecting the solar radiation intercepted [9].

VRT can be used according to the soybean management units (MU) in the field. MUs are regions within the same field that have similar characteristics and production potential [1]. Setting the different MUs can be achieved according to the past variability of the field yield, vegetation index, and/or different soil fertility and physical attribute maps [10]. In practice, establishing MUs requires on-site studies for each plot (Machado et al., 2018) and follows a trend of clustering plots with similar yield potentials. Defining potential fertility can be a complex task. There are some studies using sensors to map the apparent electrical conductivity of the soil (ECa) to help estimate this potential fertility [2], relating mainly to the soil CEC (cation exchange capacity). However, the resulting maps may vary due to soil moisture, clay content, or organic matter content [11].

Understanding the spatial variability of the soil nutritional elements can help determine the location of the MUs and assist in recommending the optimal soybean stand in the field [12]. Increased soybean stand is recommended in plots with poor soil fertility, while on plots with higher potential fertility, a reduced plant stand is required [2,9,13].

Using soil attributes to help establish MUs has the advantage of a certain temporal stability of spatial variability over different cycles in the same plot, since some soil attributes are more difficult to change over time, such as CEC, phosphorus, or clay contents [11]. The soil solution contains different amounts of different nutrients that, together with environmental factors and plant health, directly influence productivity. A mineral nutrient is an essential element for the development, quality, or yield attributes of the plant [14]. Justus Von Liebig is considered the father of plant mineral nutrition and popularized the Law of the Minimum (idealized by Carl Sprengel in 1826), by which “the yield of a crop is limited by the nutrient that is present in the least quantity in that environment” [15]. However, there are no studies relating to the use of this technique (the law of the minimum) for establishing MUs to stand variability.

The older definition of the law of the minimum in a simplistic way does not cover a more widespread and accepted current aspect, whereby many plant nutrients enhance plant growth, improve the use efficiency of nutrients, water, and other resources, increase tolerance to abiotic or biotic stress, or improve the quality or nutritional value of the harvested product [14]. Thus, the use of this principle in establishing adequate levels of nutritional elements for plants could be extended to other environmental variables that affect the soybean grain yield, including the plant stand. Therefore, hypothetically, could the variability of the optimal soybean stand be influenced by the variability of the limiting factor for maximizing the expression of the crop yield in the environment?

Seeking answers to this question, this study aimed to (i) evaluate the relationship between soil physico-chemical attributes to determine potential MUs for soybean VRT; (ii) propose a method for varying the soybean stand based on Liebig’s law of minimum; and (iii) relate the interaction between different plant stands on soybean grain yield, taking into account the spatial variability of the mapped attributes.

## 2. Materials and Methods

### 2.1. Experimental Fields

**Figure 1:**
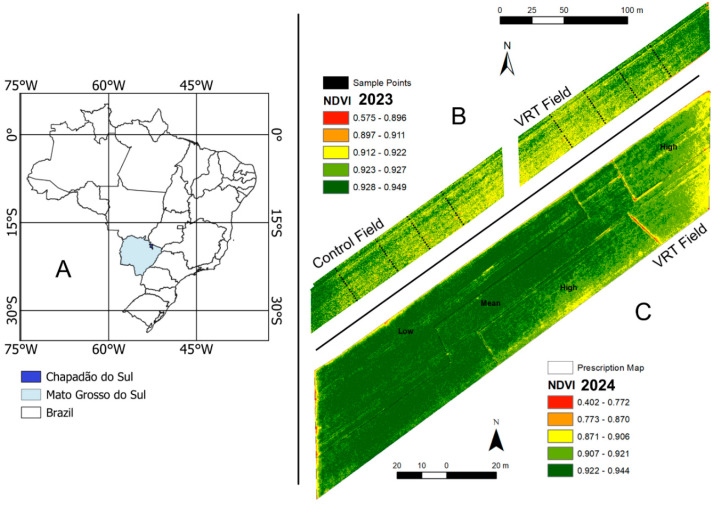
Location of the experimental fields (**A**), Normalized Difference Vegetation Index (NDVI) from the Variable Rate Technology (VRT), and Control Fields during the first crop season (**B**), and during the second crop season (**C**).

Field experiments were carried out during the 2023 and 2024 crop seasons in two agricultural areas side by side at the Federal University of Mato Grosso do Sul, municipality of Chapadão do Sul, Mato Grosso do Sul, Brazil. The two experimental areas with 1 ha each (Figure 1) differed in variable rate seeding (VRT—Variable Rate Technology Field), and a conventional seeding stand was set as a control area (Control Field). The soil in these experimental areas is classified as dystrophic red oxisol, typical of most soils in the Cerrado Biome, characterized by a high degree of weathering and low-activity clay [16]. Soil texture in the experimental field is clayey (from 415 to 490 g kg^−1^). Both experimental fields were cultivated in a minimum tillage system and had similar ranges of soil clay content.

**Figure 2:**
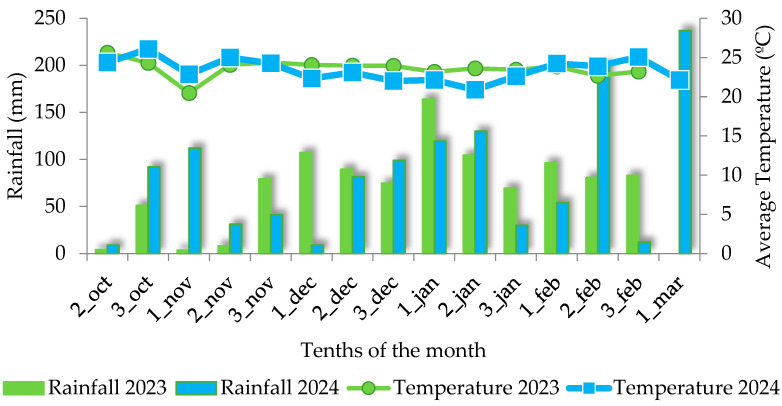
Rainfall (mm) and average temperature (°C) in ten-day periods in each month during soybean cultivation in the two experimental periods (crop seasons 2023 and 2024).

Data on climatic conditions (Figure 2) were obtained from an automated station of the National Meteorological Institute (INMET), installed in the same municipality where the experiment was carried out (18°48′ S and 52°36′ W). According to Koppen’s classification [17], the climate of the study sites is humid tropical, characterized by a rainy season during the summer and a dry season during the winter. Each year, soybeans were harvested as the principal crop, followed by corn as the second harvest of the same agricultural year. There was no climatic anomaly that could have been a restrictive factor for soybean crop yield in the two crop seasons (2023 and 2024). Rainfall was suitable for the demands of the crop.

### 2.2. Mapping the Soil and Identifying the Most Limiting Nutrients

Chemical and physical attributes of the soil in the experimental plots were measured by point sampling (with eight sub-samples) on a regular 20 × 20 m square grid at a depth of 0–0.2 m. The following attributes were assessed: clay content, macronutrients, micronutrients; base saturation (V%); pH; and cation exchange capacity (CEC). The soil analysis procedures described in [18] were used. The spatial variability of the soil apparent electrical conductivity (ECa) was also mapped using the Falker Terran model, at a depth from 0 to 0.2 m. Readings were taken at an acquisition rate of 1 s, representing one point every 5 m by walking the equipment in lines every 15 m over the field. Application of fertilizers and correctives was directly related to the yield response of the plants [19].

Geostatistical analysis and mapping were carried out using ESRI ArcGIS version 10.6.1 software, and the precepts of spatial continuity were followed to create the variograms [20]. The maps of all the mapped attributes were drawn up using the ordinary kriging interpolation method, as described by Yamamoto and Landim (2013) [21]. Modeled variograms were chosen based on the lowest mean standard error calculated by the cross-validation procedure [20].

According to the law of the minimum [15] and following the nutritional requirements to achieve the expected yield (4500 kg ha^−1^) of the soybean crop [16,19,22], the most limiting soil nutrient in the experimental field conditions was potassium (K) (Table 1). This nutrient was below the recommended level for the crop with the greatest negative discrepancy in relation to the proper levels of the nutrient. Therefore, the spatial variability of K levels in the soil, together with the CEC [2], was used to determine the different soybean populations in the second experimental year. Soybean cultivation in the first experimental year under the same plot conditions was used to test this hypothesis.

### 2.3. Experimental Design and Treatments

Soybeans were sown in November of each agricultural year, according to the local recommendation (State of Mato Grosso do Sul) for the genetic material. The soybean cultivar used was 73I75RSFIPRO (Don Mario Sementes), which belongs to maturity group 7.2 and has an indeterminate growth habit [23]. It is true that choosing an earlier or later cultivar can influence crop yields [24]. However, this proposal has to be started from scratch, as little research has been carried out to date on soybean VRT in the same field.

In the first experimental year (2023 crop season), sowing was established with different populations randomized in strips over the two fields to assess the interaction between low K levels and grain yield of each plant stand tested. Therefore, the stands were randomized over the natural variability of the soil in each field (VRT and control fields) (Figure 1B). The experimental design was completely randomized with 15 seeding strips (5 strips per treatment). Twenty-four sampling points (replications) were established for each treatment. Stand variations were as follows: very low—170,000 seeds ha^−1^; low—210,000 seeds ha^−1^; medium (recommended for the cultivar)—240,000 seeds ha^−1^; high—270,000 seeds ha^−1^; and very high—310,000 seeds ha^−1^.

The seeder used was a Jumil Pop model, with 0.50 m spacing between seeding rows. This seeder has a vertical vacuum disk seed metering system. Changing the gear ratios of the seeder was necessary to vary the plant stand when installing each treatment and in each experimental strip.

Lime was applied in September in both years using 1200 kg ha^−1^, raising the soil base saturation to 55% [16]. Fertilization at sowing was carried out with NPK (4-14-8) fertilizer at a rate of 400 kg ha^−1^, applied in the furrow by the seeder. At phenological stage V4, top dressing was carried out using potassium chloride (60% K) at a rate of 100 kg ha^−1^ [23]. Phytosanitary treatments and agricultural inputs were applied over the crop’s development according to the crop monitoring and the standards for pest control and disease in the region.

**Figure 3:**
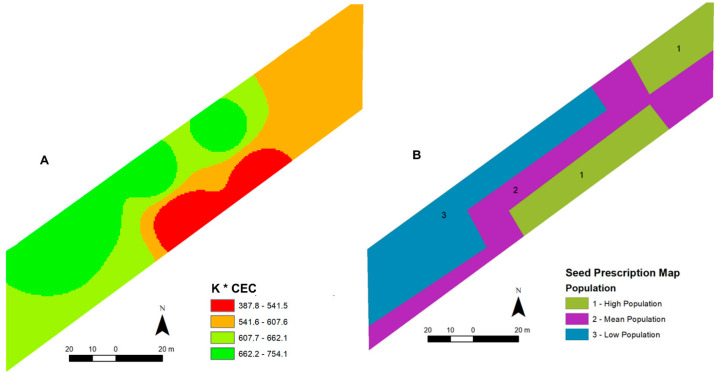
Spatial variability of the map resulting from the algebra between the K map and the CEC map (**A**) and the soybean seed prescription map (**B**).

In the second experimental year (2024 crop season), a field experiment was carried out to evaluate the variable stand based on the most limiting soil attribute considering the soybean nutritional requirements. The outcomes were compared to those of the control field (opposite), where the fixed population (220,000 plants ha^−1^), recommended for the cultivar, crop season, and growing site, was sown. After analyzing the findings from the previous experimental season, three plant populations were installed in the 2024 season with regard to what was recommended for the cultivar. The field cultivated with the VRT stand was seeded with populations varying by 15% compared to the recommended seeding rate [9]: low—190,000 seeds ha^−1^; medium—220,000 seeds ha^−1^; and high—250,000 seeds ha^−1^. The sown areas of each management unit (MU) were: low population with 3216.08 ha; medium with 3918.37 ha; and high with 2865.56 ha. The highest soybean population was sown where there was the highest limitation (K—low soil fertility level) for crop development, according to the law of the minimum and potential productivity defined by CEC [2,9,13], since potential fertility (CEC) alone is not enough to define whether there are any nutrients in the soil that are inappropriate for the crop to express its full productive potential. Based on this proposition, the soybean seed prescription map was drawn up based on the spatial variability resulting from map algebra by multiplying the K map (limiting soil nutrient in the field) and the CEC (potential fertility) (Figure 3).

### 2.4. Phenological Evaluation of the Crop in Both Crop Seasons

In both experimental years, when the soybean crop was at R5, coinciding with the beginning of grain filling [23], flights were made over the area using the SenseFly eBee RTK unmanned aerial vehicle (UAV). This device was equipped with a Parrot Sequoia multispectral camera to take images, which enabled the crop vegetation mass to be inferred [1] and correlated with the other experimental variables. Radiometric calibration of the images was carried out. Orthorectification was carried out using Pix4D software version 4.1.22. Multispectral reflectance images were obtained for the green (550 nm ± 40 nm), red (660 nm ± 40 nm), red-edge (735 nm ± 10 nm), and near-infrared (NIR, 790 nm ± 40 nm) spectral bands, allowing the most commonly used vegetation indices (IV) to be calculated: NDVI, SAVI, NDRE, and GNDVI [1]. Overflights were carried out at 100 m local altitude, allowing for a spatial resolution of 0.10 m in the image. Plant stand was assessed in the plots at 25 days after emergence (DAE), at each sampling point, when the soybean crop was at phenological stage V4 [23]. After the overflights, when the crop was at phenological stage R5, two experimental variables were measured: plant height (m), measured with a tape measure from ground level; and the fresh weight of the plants (g) on the sampling points, determined by the average mass of three plants cut off close to the ground, measured with a hook-type digital scale, and under field conditions.

The soybeans were harvested by hand from the same sampling points in each crop season, and the soybeans from the 2 m^2^ point sampling area were harvested and threshed. Data were filtered, normalized, and the map was obtained by interpolation. After harvesting, the variables of 100-grain mass (g) and number of pods per plant were also measured.

### 2.5. Statistical Analysis

An analysis of variance was applied to the treatments to check the level of significance and compare the yield between the treatments. Pearson’s correlation network was drawn up to graphically express the relationship between the treatments, in which the proximity between the nodes (traces) is proportional to the absolute value of the correlation between these nodes [25]. Subsequently, the analysis of canonical variables (multivariate statistics) was carried out to verify the interrelationship between the variables evaluated. Some variables that stood out were also analyzed individually by Duncan’s mean comparison test at 5% probability. Analyses were carried out using RBio software [26] (https://www.r-project.org/).

## 3. Results

### 3.1. The First Experimental Year

Figure 1A shows the correlation network between the variables analyzed for the first experimental year (random strip sowing). It can be seen that grain yield showed a positive relationship (thicker green traces) with the K level in the soil and with the NDVI vegetation index. It also showed a direct relationship with the NDRE index, but with a lower correlation (thinner green traits). The fresh plant weight showed a more significant correlation with the NDRE, but it was not statistically significant in relation to yield.

The 100-grain weight also showed no significant relationship with yield, most likely because it is a phenological variable related to the genetics of the plant material [23]. Apparent soil electrical conductivity (ECa) showed a positive and significant correlation with cation exchange capacity (CEC), agreeing with [11]. Both variables did not show any relationship with crop productivity. The experimental plot in question presented high levels of P (Table 1), so it was not an element that proved to be limiting for productivity, even leading to a negative relationship (red line) between P and productivity. Variations in K and P levels are common in Brazilian Cerrado soils and may occur because of continuous fertilizer application at a fixed rate of nutrients over the agricultural years [27], and since the P has practically no mobility in the soil, annual fertilizations eventually increase the levels of this element. These relationships found in the first experimental year may be due to the fact that the soybean was sown randomly in strips, not considering the spatial variability of the nutrients in the soil or the spatial variability of the CEC.

**Figure 4:**
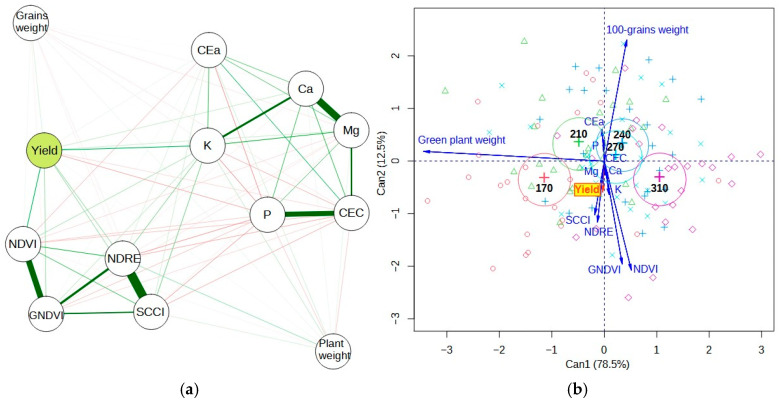
Pearson’s correlation network (**a**) between the yield variables grain yield, 100-grain weight, and green plant weight; soil variables apparent soil electrical conductivity (ECa), cation exchange capacity (CEC), calcium (Ca), magnesium (Mg), potassium (K), and phosphorus (P) levels; and vegetation indices NDVI, NDRE, SCCI, and GNDVI, evaluated in the first experimental year. Canonical variable analysis (**b**) as a function of plant stands varying between 170,000 plants ha^−1^ (170) and 310,000 plants ha^−1^ (310), evaluated in the first experimental year. In (**a**) green lines indicate positive correlations, while red lines indicate negative correlations.

Canonical variable analysis (Figure 4b) explained most of the variability in the data, as the sum of the first two canonical variables was 91%, considerably higher than the minimum acceptance level of 70% [25]. This technique uses the residual covariance between variables to generate a biplot. Soybean crop yield showed variability associated with plant stands and K levels in the soil (proximity and size between yield and K vectors), even with the experiment being sown on random strips with different stands. The proximity between the vectors of vegetation indices indicates that they showed similar variability in their results. The variation in soybean stands across the different treatments (170 and 310 thousand plants per hectare) affected the variation in crop yield. We found that the average weight of the green plants was inversely proportional to the population density, reflecting the higher mass in the treatments where the seeding rate was lower compared to the treatments with the highest density of plants per hectare. The analysis of crop yield variation in treatments with intermediate plant stands showed higher stability of this response variable in these treatments when compared to the maximum and minimum stands, which showed higher variability.

**Figure 5:**
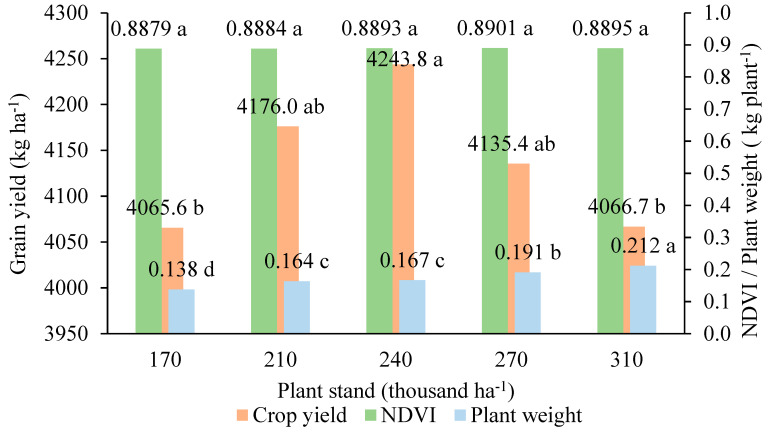
Relationship between soybean grain yield in the first experimental year (strip sowing), NDVI levels, and plant green mass, according to the different plant stands. Equal letters in each response variable represent statistical similarity, according to Duncan’s test at 5% probability.

Thus, even disregarding the spatial variability of soil CEC and K nutrient levels (sowing the first experimental year in random strips), soybean yields were variable and dependent on the different plant stans (Figure 5). There was no variation in NDVI levels between the plant stands. However, there was variation in the green mass of the plants. As plant stand variation increased, plant mass also increased. We noted on the field that intraspecific competition between the plants changed their architecture, causing them to grow in a stiolated plant, which reflected on the green mass. This competition affects the formation of branches, directly influencing the interception of solar radiation [9]. In this way, the plant can develop greater mass, but this does not necessarily result in increased yield. Highest soybean yields were achieved at intermediate stands (210 and 270 thousand plants ha^−1^), and so the soybean stand variations were established in the second experimental year (VRT experiment), when the stand variation followed the spatial variability of K and CEC. Stand variations were adopted, varying by 15% in relation to that recommended for the crop under local conditions [9].

Soybean crops are able to compensate for the lower plant stand by growing new branches [9]. However, if the population density is reduced excessively, the crop may not be able to reach the critical leaf area index, and the resulting grain yield may decrease. On the other hand, by increasing the population density, it is possible that the critical leaf area index will be reached quickly in the early crop stages, which could even lead to a decrease in yield due to excessive intraspecific competition [28], as well as higher implementation costs due to higher seed costs.

### 3.2. The Second Experimental Year

**Figure 6:**
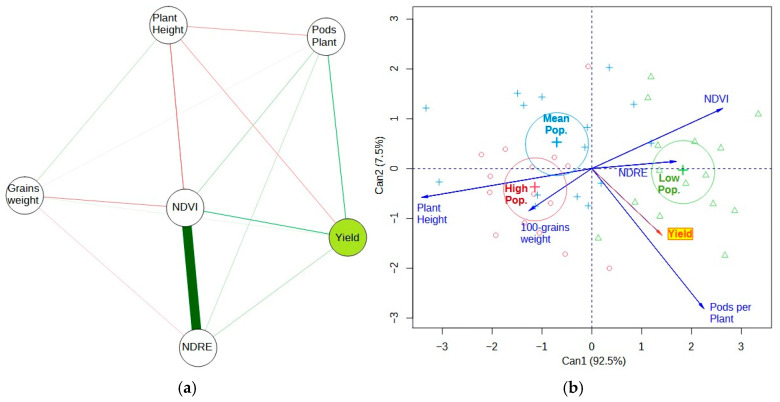
Pearson’s correlation network (**a**) between the yield variables 100-grain weight, pods per plant, plant height and grain yield, and vegetation indices NDVI and NDRE, evaluated in the second experimental year. Canonical variable analysis (**b**) as a function of plant stands varying between 190,000 plants ha^−1^ (Low pop.), 220,000 (Mean pop.), and 250,000 plants ha^−1^ (High pop.), evaluated in the second experimental year. In (**a**) green lines indicate positive correlations, while red lines indicate negative correlations.

Figure 6a shows the Pearson correlation network between the variables analyzed in the second experimental year (VRT). It can be seen that the grain yield showed a positive relationship with the other response variables, NDVI and pods per plant. However, crop yield was inversely proportional to plant height. The 100-grain weight showed no statistically significant relationship with yield variability, corroborating the results found in the first experimental year. The inclusion of the soil variables was not justified in this analysis since the statistical treatments (variation in VRT) were installed due to the spatial variability of these conditions. Canonical variable analysis (Figure 6b) shows that the variability of the VRT treatments influenced the variation in crop yield, especially in the lower plant stand treatment. The highest plant stand affected the variability of the plant height. The variability in the number of pods per plant is closely and positively correlated with crop yield. Thus, areas with lower plant stands had higher numbers of pods per plant and higher yields. Furthermore, the variability of the NDVI was inversely proportional to plant height, according to the soybean plant stand established in VRT.

**Figure 7:**
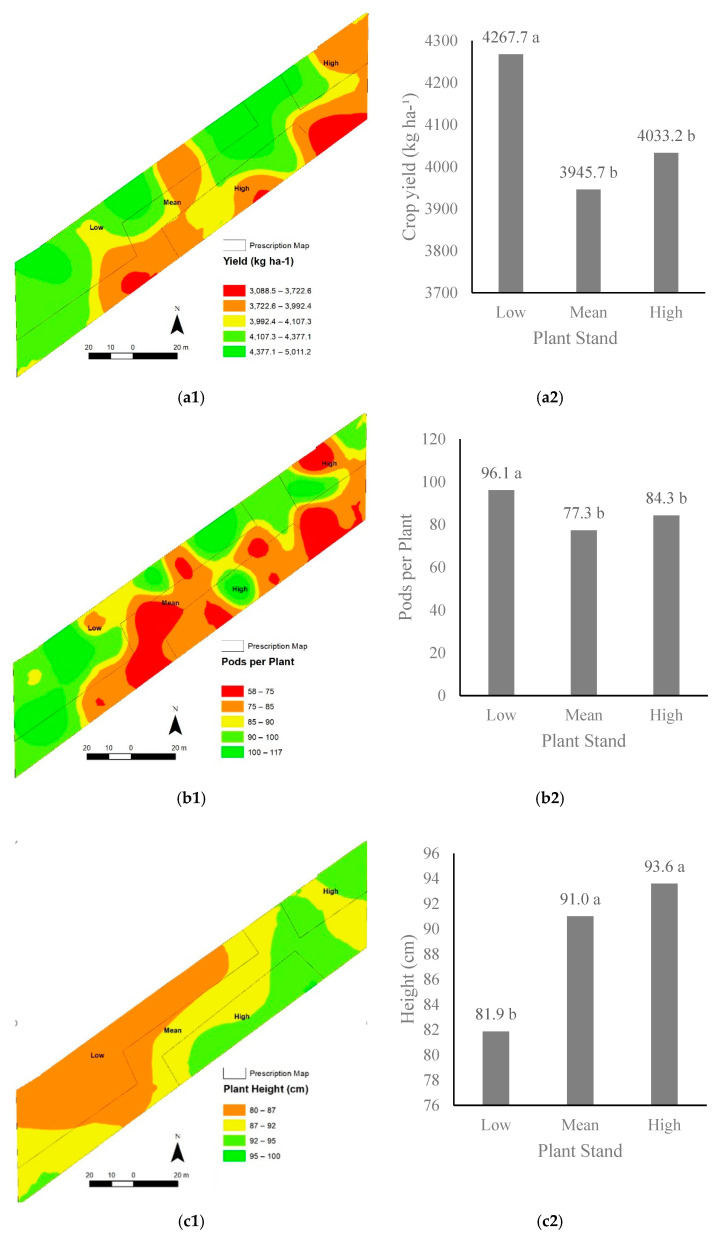
Spatial variability and means of yield (**a1**,**a2**, respectively), pods per plant (**b1**,**b2**, respectively), and plant height (**c1**,**c2**, respectively) in the different management units seeded with soybean plant stand variation (low, mean, and high populations), and comparison of the means for these variables. Equal letters represent statistical similarity by Duncan’s test at 5% probability.

Figure 7 shows the spatial variability maps of yield, pods per plant and plant height resulting from VRT, following the precision agriculture principles, in the different management units. Yields in the different treatments were statistically different and were highest when the crop was seeded with the lowest plant population stand. Thus, by seeding the lowest population of soybean plants in the management units with the least limitations (higher K availability and higher CEC level) for crop development, it was possible to achieve the highest yields with the lowest investment in seeds. This region also had a higher number of pods per plant (Figure 7b2) and lower plant height (Figure 7c2). Management units that received the highest soybean plant stand showed high intraspecific competition, resulting in higher plant height and lower yields. This management unit showed statistically similar results to the management unit that received the median stand. Therefore, increasing the soybean stand (according to the recommendations of the genetic material developers) in management units where fertility conditions are more limited is not interesting, as there will be a higher cost for the seed input and there will be no benefit in terms of increased yield; conversely, there will be a higher plant height, which could lead to the crop becoming lodged or a greater risk of leaf disease incidence. The mean yield of the soybean crop measured in the VRT plot was 4100.7 kg ha^−1^, while the mean yield of the control plot (fixed seeding rate) was 3720.3 kg ha^−1^, which is 9.7% lower than the mean yield achieved in the plot sown according to the precision agriculture principles (VRT seeding).

## 4. Discussion

Variation in soybean plant stand reflecting on grain yield has already been explored in previous studies [1,3,9,12], but the question that still needs to be answered is: “Where exactly and based on what factors should the plant stand vary?” As presented in the experimental results, it is possible to follow the principles of the law of minimum as a starting point for answering this question. Variable-rate sowing of annual crops has been investigated in recent years. However, to the best of our knowledge, there are no similar studies in the literature. Moura et al. [2] proposed a methodology for soybean seeding at variable rates based on soil electrical conductivity. The authors concluded, based on a single harvest, that increasing the soybean seed population by 20% in locations with lower electrical conductivity and decreasing the seed population by 20% in locations with higher electrical conductivity was effective and resulted in increased grain yield. Silva et al. [29] state that maximum corn productivity can be achieved by varying the seed population according to soil attributes, and within soil attributes they proved that magnesium and apparent electrical conductivity were the most important in determining plant stand. Silva et al. [13] used apparent electrical conductivity and soil clay content to perform variable-rate soybean sowing and observed significant increases in grain yield using the proposed methodology. In the literature, there are no studies that use the most limiting soil element, i.e., the law of minimum, to define plant stand.

Plant stand is one of the main factors to be managed in order to optimize soybean yields [30]. However, in situations where soybeans are seeded with the same plant stand, not taking into account the spatial variability of the soil elements that determine crop yield, as was shown in the results of the first experimental year (random strip seeding), the median stand recommendation is more appropriate since it tends to lead to the highest yields. This is due to the fact that in situations where the limiting nutrient for crop yield is not identified or the spatial variability of this element is not even mapped, the spatial variability conditions for yield become random, and hence it would be a mistake to guide stand variation without knowing what the limiting condition is for the plant to achieve maximum yield.

Ideal plant stands for soybean crops will depend on factors mainly related to nutrient availability, water status, and photosynthetically active radiation available and can vary from year to year, mainly due to cyclical climatic events such as El Niño [31]. When there is no water or nutritional limitation, the growth of a crop will be directly related to the amount of photosynthetically active radiation intercepted by the canopy and the efficiency at which this radiation is transformed into dry matter [5]. Therefore, seeding density can directly affect the radiation intercepted. Soybean plant weight and height increased with higher plant stands. It is very likely that this variation was due to the plant etiolation, resulting in an attempt to increase leaf area and higher photosynthetic energy interception by the plant as a result of intraspecific competition.

By reducing the plant stand in regions with higher fertility soils, there was a reduction in plant height, an increase in the number of pods per plant, and an increase in grain yield. Therefore, it is possible to reduce the soybean plant stand in management units where there are no nutritional restrictions, also reducing the cost of investing in seeds. Ref. [9] reported that the soybean seeding rate can be reduced by up to 18% in management units where historical yields are high (zones with higher yield potential) compared to management units with low yield potential, without penalizing yields. However, there is some difficulty in establishing management units based on the variability of soybean yields since these maps have great dissimilarity between years [13]. A rather different situation for the maize crop.

Using the CEC map alone does not explain the yield variability on its own and should not be used as a guide (because CEC is potential, not the actual nutrient availability status). The K variability map (as the most limiting nutrient) explained much more of the variability in yield than the soil CEC. The K nutrient, after N, is the most demanded by the soybean crop. This nutrient is exported at a rate of 20 kg per ton of grain produced by the crop [19]. K has several important metabolic functions in the plant, such as activating enzymes, synthesizing starch and proteins, regulating osmotic potential, and opening and closing stomata [22]. Recommendations for potassium fertilization and appropriate levels in the soil, especially in the Cerrado region, have been changing recently, as genetically improved cultivars are achieving yields above 5000 kg ha^−1^. Traditional and outdated studies that focused on potassium fertilization in the Cerrado date back to a time when the mean expected yield for the soybean crop was 3600 kg ha^−1^ ha^−1^ [16]. Therefore, there is a widespread consensus that the K levels in the soil should be adjusted according to the recent plant breeding that has been carried out on the crop to ensure a higher expected yield [19].

Increasing the soybean population (in relation to that recommended by the developers of the genetic material) in management units with more restricted fertility is not interesting, as there will be a higher cost for the seed input and there will be no benefit in increasing grain yield. In contrast, there will be a higher plant height, which could lead to the crops becoming lodged or a greater risk of leaf disease incidence.

Future studies should focus on the physiological mechanisms underlying the major production factors (soil–water–plant relations), which contribute to the crop yield response, aiming at improving the accuracy of soybean seeding rate recommendations for management units. Applying the law of minimum to recommend plant stands serves to optimize fertilizer management in soybean. The law of minimum states that plant growth is limited by the scarcest resource, and it is essential to identify which nutrients or soil conditions represent the main limiting factors for grain yield. By adjusting fertilizer management to address these specific deficiencies, it is possible to avoid excessive use of inputs, reduce costs, and mitigate environmental impacts while maximizing the productive potential of soybeans.

## 5. Conclusions

Management units can be established according to the precision agriculture principles for soybean variable-rate seeding, following the spatial variability of soil nutrients that are limiting crop yield potential according to the law of the minimum and thus increasing the average grain yield. The spatial variability of soil K in the plot (specific condition of this particular experimental plot), which was identified as limiting nutrients according to the law of the minimum, affected the spatial variability of soybean yields. Further studies should be carried out in each plot to identify the nutrients in a limiting condition before establishing management units.

Reducing the plant stand in management units with lower limitations for crop development increases grain yield in these areas. However, increasing the population of soybean plants in management units with a lower nutritional level, or a higher level of limitation, can lead to increased intraspecific competition, affecting grain yield in these areas, as well as higher input demand.

## Figures and Tables

**Table 1:** Descriptive statistical analysis of measured values for the soil attributes and nutrient level interpretation.

	ph	Ca	Mg	K	P	Cu	Fe	Mn	Zn	CEC	V	Clay
	-	cmol dm^−3^		mg dm^−3^						cmol dm^−3^	%	
Mean	5.3	4.1	1.4	66.5	29.8	1.8	66.4	16.5	7.9	9.1	61.6	46.1
Nutrient Level	A	A	A	L	H	H	H	H	H	-	A	-
Minimum	4.8	3.0	0.8	44.0	14.0	0.9	52.0	10.9	5.0	8.5	44.4	41.5
Maximum	5.7	5.2	2.0	81.0	56.9	2.8	82.0	20.7	10.8	9.9	75.2	49.0
Coeff Variation	5.9%	17.1%	29.2%	18.2%	45.5%	31.7%	14.5%	21.4%	25.4%	4.7%	16.8%	5.0%
Adequate	5.2	4.3	1.3	130.0	15.0	0.8	30.0	5.0	1.6	-	50.0	-
Discrepance	1.0%	−4.0%	9.9%	−95.5%	47.9%	54.6%	54.8%	69.7%	79.8%	-	18.8%	-

L (low); A (adequate); H (high); CEC (cation exchange capacity); and V (base saturation).

## Data Availability

The original contributions presented in this study are included within the article; further inquiries can be directed to the corresponding author.

## References

[1] da Silva E.E., Rojo Baio F.H., Ribeiro Teodoro L.P., da Silva Junior C.A., Borges R.S., Teodoro P.E. (2020). UAV-Multispectral and Vegetation Indices in Soybean Grain Yield Prediction Based on in Situ Observation. Remote Sens. Appl..

[2] Moura S.S., França L.T., Pereira V.S., Teodoro P.E., Baio F.H.R. (2020). Seeding Rate in Soybean According to the Soil Apparent Electrical Conductivity. An. Acad. Bras. Cienc..

[3] Correndo A., McArtor B., Prestholt A., Hernandez C., Kyveryga P.M., Ciampitti I.A. (2022). Interactive Soybean Variable-Rate Seeding Simulator for Farmers. Agron. J..

[4] Thrash B.C., Catchot A.L., Gore J., Cook D., Musser F.R., Irby T., Krutz J. (2021). Effects of Soybean Plant Population on Yield Loss From Defoliation. J. Econ. Entomol..

[5] Murchie E.H., Burgess A.J. (2022). Casting Light on the Architecture of Crop Yield. Crop Environ..

[6] Wang Q., Bai W., Sun Z., Zhang D., Zhang Y., Wang R., Evers J.B., Stomph T., van Der Werf W., Feng C. (2021). Does Reduced Intraspecific Competition of the Dominant Species in Intercrops Allow for a Higher Population Density?. Food Energy Secur..

[7] Zhang W.-P., Liu G.-C., Sun J.-H., Zhang L.-Z., Weiner J., Li L. (2015). Growth Trajectories and Interspecific Competitive Dynamics in Wheat/Maize and Barley/Maize Intercropping. Plant Soil.

[8] Wang Y., Zhao Z., Li J., Zhang M., Zhou S., Wang Z., Zhang Y. (2017). Does Maize Hybrid Intercropping Increase Yield Due to Border Effects?. Field Crops Res..

[9] Carciochi W.D., Schwalbert R., Andrade F.H., Corassa G.M., Carter P., Gaspar A.P., Schmidt J., Ciampitti I.A. (2019). Soybean Seed Yield Response to Plant Density by Yield Environment in North America. Agron. J..

[10] Albornoz E.M., Kemerer A.C., Galarza R., Mastaglia N., Melchiori R., Martínez C.E. (2018). Development and Evaluation of an Automatic Software for Management Zone Delineation. Precis. Agric..

[11] Molin J.P., Tavares T.R. (2019). Sensor Systems for Mapping Soil Fertility Attributes: Challenges, Advances, and Perspectives in Brazilian Tropical Soils. Eng. Agrícola.

[12] Machado M.V., Maggi M.F., Souza E.G.D., Camicia R.G.D.M., Amarante R.R.D. (2018). Application of Plant Densities in Management Units in the Soybean Cultivation. J. Agric. Sci..

[13] da Silva E.E., Baio F.H.R., Teodoro L.P.R., Campos C.N.S., Plaster O.B., Teodoro P.E. (2022). Variable-Rate Seeding in Soybean According to Soil Attributes Related to Grain Yield. Precis. Agric..

[14] Brown P.H., Zhao F.-J., Dobermann A. (2022). What Is a Plant Nutrient? Changing Definitions to Advance Science and Innovation in Plant Nutrition. Plant Soil..

[15] Tang J., Riley W.J. (2021). Finding Liebig’s Law of the Minimum. Ecol. Appl..

[16] Sousa D.M.G., Lobato E. (2004). Cerrado: Soil Correction and Fertilization.

[17] Alvares C.A., Stape J.L., Sentelhas P.C., Gonçalves J.D.M., Sparovek G. (2013). Köppen’s Climate Classification Map for Brazil. Meteorol. Z..

[18] Teixeira P.C., Donagemma G.K., Fontana A., Teixeira W.G. (2017). Manual de Métodos de Análise de Solo.

[19] Oliveira Junior A., Castro C., Oliveira F.A., Klepker D., Seixas C.D.S. (2020). Soil Fertility and Assessment of Soybean Nutritional Status. Soybean Production Technologies.

[20] Yamamoto J.K., Landim P.M.B. (2013). Geostatistics. Concepts and Applications.

[21] Yamamoto S., Nomoto S., Hashimoto N., Maki M., Hongo C., Shiraiwa T., Homma K. (2023). Monitoring Spatial and Time-Series Variations in Red Crown Rot Damage of Soybean in Farmer Fields Based on UAV Remote Sensing. Plant Prod. Sci..

[22] Moreira A., Moraes L.A.C., Prado R.d.M., Campos C.N.S. (2018). Nutrition and Fertilization of Soybean Crops: Macronutrients. Nutrition and Fertilization of Large Crops.

[23] Neumaier N., Farias J.R.B., Nepomuceno A.L., Mertz-Henning L.M., Foloni J.S.S., Moraes L.A.C., Gonçalves S.L., Seixas C.D.S. (2020). Soybean Ecophysiology. Soybean Production Technologies.

[24] Kruk B., Satorre E.H., Satorre E.H. (2003). Density and Spatial Arrangement of Cultivation. Grain Production: Functional Bases for Its Management.

[25] Bhering L.L., Teodoro P.E. (2021). Experimental Statistics at Rbio.

[26] Bhering L.L. (2017). Rbio: A Tool for Biometric and Statistical Analysis Using the R Platform. Crop Breed. Appl. Biotechnol..

[27] de Oliveira L.E.Z., de Souza Nunes R., de Sousa D.M.G., de Figueiredo C.C. (2020). Dynamics of Residual Phosphorus Forms under Different Tillage Systems in a Brazilian Oxisol. Geoderma.

[28] Ebone L.A., Caverzan A., Tagliari A., Chiomento J.L.T., Silveira D.C., Chavarria G. (2020). Soybean Seed Vigor: Uniformity and Growth as Key Factors to Improve Yield. Agronomy.

[29] da Silva E.E., Baio F.H.R., Kolling D.F., Júnior R.S., Zanin A.R.A., Neves D.C., Fontoura J.V.P.F., Teodoro P.E. (2021). Variable-Rate in Corn Sowing for Maximizing Grain Yield. Sci. Rep..

[30] Souza R., Teixeira I., Reis E., Silva A. (2016). Soybean Morphophysiology and Yield Response to Seeding Systems and Plant Populations. Chil. J. Agric. Res..

[31] Nwokolo S.C., Meyer E.L., Ahia C.C. (2024). Exploring the Interactive Influences of Climate Change and Urban Development on the Fraction of Absorbed Photosynthetically Active Radiation. Atmosphere.

